# Anti-influenza virus effect of aqueous extracts from dandelion

**DOI:** 10.1186/1743-422X-8-538

**Published:** 2011-12-14

**Authors:** Wen He, Huamin Han, Wei Wang, Bin Gao

**Affiliations:** 1 CAS Key Laboratory of Pathogenic Microbiology and Immunology (CASPMI), Institute of Microbiology, Chinese Academy of Sciences, 1 Beichen West Road, Beijing 100101, PR China; 2 Graduate University of Chinese Academy of Sciences, 1 Beichen West Road, Beijing 100101, PR China; 3 Biochemistry Teaching and Research office of Hebei Medical University, Zhongshan East Road, Shijiazhuang 050017, PR China; 4 China-Japan Joint Laboratory of Molecular Immunology and Microbiology, Institute of Microbiology, Chinese Academy of Sciences, Beijing, PR China

**Keywords:** Dandelion, Anti-influenza virus, Traditional Chinese Medicine

## Abstract

**Background:**

Human influenza is a seasonal disease associated with significant morbidity and mortality. Anti-flu Traditional Chinese Medicine (TCM) has played a significant role in fighting the virus pandemic. In TCM, dandelion is a commonly used ingredient in many therapeutic remedies, either alone or in conjunction with other natural substances. Evidence suggests that dandelion is associated with a variety of pharmacological activities. In this study, we evaluated anti-influenza virus activity of an aqueous extract from dandelion, which was tested for in vitro antiviral activity against influenza virus type A, human A/PR/8/34 and WSN (H1N1).

**Results:**

Results obstained using antiviral assays, minigenome assay and real-time reverse transcription-PCR analysis showed that 0.625-5 mg/ml of dandelion extracts inhibited infections in Madin-Darby canine kidney (MDCK) cells or Human lung adenocarcinoma cell line (A549) of PR8 or WSN viruses, as well as inhibited polymerase activity and reduced virus nucleoprotein (NP) RNA level. The plant extract did not exhibit any apparent negative effects on cell viability, metabolism or proliferation at the effective dose. This result is consistent with the added advantage of lacking any reported complications of the plant's utility in traditional medicine over several centuries.

**Conclusion:**

The antiviral activity of dandelion extracts indicates that a component or components of these extracts possess anti-influenza virus properties. Mechanisms of reduction of viral growth in MDCK or A549 cells by dandelion involve inhibition on virus replication.

## Background

Influenza A viruses are negative strand RNA viruses with a segmented genome that belong to the family of orthomyxoviridae. Both influenza A and B viruses can infect humans and cause annual influenza epidemics which result in significant mobidity and mortality worldwide. There are 16 hemagglutinin (HA) and 9 neuraminidase (NA) subtypes of the influenza A virus that infect a wide variety of species [1]. The introduction of avian virus genes into the human population can happen at any time and may give rise to a new pandemic. There is even the possibility of a direct infection of humans by avian viruses, as evidenced by the emergence of the highly pathogenic avian influenza viruses of the H5N1 subtype that were capable of infecting and killing humans [2].

Vaccines are the best option for the prophylaxis and control of a pandemic; however, the lag time between virus identification and vaccine distribution exceeds 6 months and concerns regarding vaccine safety are a growing issue leading to vaccination refusal. In the short-term, antiviral therapy is vital to control the spread of influenza. To date, only two classes of anti-influenza drugs have been approved: inhibitors of the M2 ion channel, such as amantadine and rimantadine, or neuraminidase inhibitors, such as oseltamivir or zanamivir [3]. Treatment with amantadine, and its derivatives, rapidly results in the emergence of resistant variants and is not recommended for general or uncontrolled use [4]. Among H5N1 isolates from Thailand and Vietnam, 95% of the strains have been shown to harbor genetic mutations associated with resistance to the M2 ion channel-blocking amantadine and its derivative, rimantadine [5]. Furthermore, influenza B viruses are not sensitive to amantadine derivatives [6]. Recent studies have reported that the development of resistance can also occur against neuraminidase inhibitors [7]. According to a recent study, oseltamivir-resistant mutants in children being treated for influenza with oseltamivir appear to arise more frequently than previously reported [8]. In addition, there are several reports suggesting that resistance in H5N1 viruses can emerge during the currently recommended regimen of oseltamivir therapy and that such resistance may be associated with clinical deterioration [9]. Thus, it has been stated that the treatment strategy for influenza A (H5N1) viral infections should include additional antiviral agents. All these highlight the urgent need for new and abundantly available anti-influenza agents.

A number of anti-flu agents have been discovered from Traditional Chinese Medicine (TCM) herbs. Ko et al. found that TCM herbal extracts derived from *Forsythia suspensa *('Lianqiao'), *Andrographis paniculata *('Chuanxinlian'), and *Glycyrrhiza uralensis *('Gancao') suppressed influenza A virus-induced RANTES secretion by human bronchial epithelial cells [10]. Mantani et al. reported that the growth of influenza A/PR/8/34 (H1N1) (PR8) virus was inhibited when the cells were treated with an extract of *Ephedraspp *('Mahuang') [11]. Hayashi et al. found that trans-cinnamaldehyde of *Chinese cinnamon *('Rougui') could inhibit the growth of influenza A/PR/8 virus in vitro and in vivo [12]. Park et al. found that *Alpinia Katsumadai *extracts and fractions had strong anti-influenza virus activity in vitro [13]. Many TCM herbs have been found to be anti-flu agents, but their mechanisms of action have not yet been elucidated [14,15].

Plants have a long evolutionary history of developing resistance against viruses and have increasingly drawn attention as potential sources of antiviral drugs [16,17]. Dandelion belongs to the *Compositae *family, which includes many types of traditional Chinese herbs [18]. Dandelion is a rich source of vitamins A, B complex, C, and D, as well as minerals such as iron, potassium, and zinc. Its leaves are often used to add flavor to salads, sandwiches, and teas. The roots can be found in some coffee substitutes, and the flowers are used to make certain wines. Therapeutically, dandelion has the ability to eliminate heat and toxins, as well as to reduce swelling, choleresis, diuresis, and inflammation [19]. Dandelion has been used in Chinese folklore for the treatment of acute mastitis, lymphadenitis, hepatitis, struma, urinary infections, cold, and fever. Choi et al. found that dandelion flower ethanol extracts inhibit cell proliferation and induce apoptosis in human ovarian cancer SK-OV-3 cells [20]. Hu et al. detected antioxidant, pro-oxidant, and cytotoxic activities in solvent-fractionated dandelion flower extracts in vitro [21]. Kim et al. demonstrated antioxidative, anti-inflammatory and antiatherogenic effects of dandelion (*Taraxacum officinale*) extracts in C57BL/6 mice, fed on an atherogenic diet [22]. Ovadje et al. suggested that aqueous dandelion root extracts contain components that induce apoptosis selectively in cultured leukemia cells, emphasizing the importance of this traditional medicine [23]. Furthermore, there are no side effects associated with the prolonged use of dandelion for therapeutic purposes.

In this report, we attempted to analyze whether dandelion have anti-influenza virus activity in cell culture. We found dandelion could inhibit the influenza virus infection. We further identified the inhibition of viral polymerase activity and the reduction of the virus nucleoprotein (NP) RNA level contributed to the antiviral effect. Thus, dandelion may be a promising approach to protect against influenza virus infections.

## Methods

### Evaluation and extraction of plant materials

Extracts made by boiling the herb in water. The voucher specimen of the plant material was deposited in the CAS Key Laboratory of Pathogenic Microbiology and Immunology (CASPMI), Institute of Microbiology, Chinese Academy of Sciences. Dandelion, purchased from a medicine store, was dissolved in sterile H_2_O (100 mg/ml) at room temperature for 2 h and then extracted twice with water at 100°C for 1 h. The aqueous extracts were filtered through a 0.45 μm membrane. This aqueous dandelion extract lyophilized, and the resulting light yellow powder (17% w/w yield) was dissolved with cell culture medium when needed.

### Viruses, cells and viral infections

Human influenza virus A/Puerto Rico/8/34 (H1N1) (PR8) and A/WSN33 (WSN) were grown in 10-day old fertilized chicken eggs. After incubation at 37°C for 2 days, the allantoic fluid was harvested and used for infection.

All cell lines were purchased from ATCC (Rockville, MD, USA). Madin-Darby canine kidney (MDCK) cells or Human lung adenocarcinoma cell line (A549) were cultured in Dulbecco's modified eagle medium (DMEM) or RPMI-1640 medium, respectively, with 10% fetal bovine serum (FBS, Gibco, USA), penicillin 100 U/ml, and streptomycin 10 μg/ml. Prior to infection, the cells were washed with phosphate-buffered saline (PBS) and were cultured in infection medium (DMEM without FBS, 1.4% BSA) supplemented with antibiotics and 2 μg/ml of trypsin (Gibco; Invitrogen, Carlsbad, CA).

### Hemagglutination inhibition test

Influenza viruses are characterized by their ability to agglutinate erythrocytes. This hemagglutination activity can be visualized upon mixing virus dilutions with chicken erythrocytes in microtiter plates. The chicken erythrocytes were supplemented with 1.6% sodium citrate (Sigma, USA) in sterile water, separated by centrifugation (800 × g, 10 min, room temperature) and washed three times with sterile PBS. Serial two-fold dilutions of dandelion extracts were made in 25 μl of PBS in 96-well V-bottom plates. Influenza viruses in 25 μl of PBS (4 HAU) were added to each dilution, and the plates were incubated for 1 h at room temperature. 25 μl of 1% (v/v) chicken erythrocytes in PBS was added to each well. The hemagglutination pattern was read following the incubation of the plates for 0.5 h at room temperature. The highest dilution that completely inhibited hemagglutination was defined as the hemagglutination inhibition (HI) titer.

### Cell viability assay

A549 or MDCK cells were left untreated or treated with the indicated amounts of dandelion extracts ranging from 20 to 0.1563 mg/ml, and oseltamivir ranging from 12.5 to 0.098 mg/ml for 48 h; MDCK cells were left untreated or treated with 0.1 mg/ml oseltamivir, 2.5 mg/ml and 15 mg/ml dandelion extracts for 72 h. All drugs were multiproportion diluted in serum-free medium. Cell-proliferation and metabolism were measured using the CCK8-assay. Briefly, the cells were treated with CCK-8 solution (dojindo, 10 μl/well) and incubated for 4 h at 37°C. The absorbance was measured using a microplate reader (DG5032, Huadong, Nanjing, China) at 450 nm. The untreated control was set at 100%, and the treated samples were normalized to this value according to the following equation: Survival rate (%) = optical density (OD) of the treated cells - OD of blank control/OD of negative control - OD of blank control × 100.

### Plaque titrations and antiviral assays

Plaque titrations: MDCK cells grown to 90% confluency in 96-well dishes were washed with PBS and infected with serial dilutions of the supernatants in PBS for 1 h at 37°C. The inoculum was aspirated and cells were incubated with 200 μl DMEM (medium containing 1.4% BSA, 2 μg/ml of trypsin and antibiotics) at 37°C, 5% CO_2 _for 2-3 days. Virus plaques were visualized by staining with trypan blue.

Antiviral assay: MDCK cells were infected with the influenza A virus strain PR8 or WSN (1 × 10^6 ^PFU) and were left untreated or treated with dandelion extracts (0.0782-5 mg/ml), oseltamivir (0.0047-0.3 mg/ml) (Sigma), or suxiaoganmaojiaonang (0.069-4.375 mg/ml). At 16 h post infection supernatants were taken. This procedure was repeated two times in triplicate. Supernatants were assayed for progeny virus yields by standard plaque titrations. Virus yields of mock-treated cells were arbitrarily set as 100%.

Simultaneous treatment assay: dandelion extracts (2.5 mg/ml), oseltamivir (0.1 mg/ml) or suxiaoganmaojiaonang (4.375 mg/ml) was mixed with virus individually and incubated at 4°C for 1 h. The mixture was inoculated onto near confluent MDCK cell monolayers (1 × 10^5 ^cells/well) for 1 h with occasional rocking. The solution was removed, the cells were washed twice with PBS and the inoculum was aspirated, and then the cells were incubated with 2 ml of DMEM supplemented with 1.4% BSA, antibiotics, 2 μg/mL trypsin at 37°C under 5% CO_2 _atm.

Post treatment assay: Influenza viruses (1 × 10^6 ^PFU) were inoculated onto near confluent MDCK cell monolayers (1 × 10^5 ^cells/well) for 1 h with occasional rocking. The media was removed and replaced by DMEM containing 1.4% BSA, antibiotics, 2 μg/mL trypsin and dandelion extracts (2.5 mg/ml), or oseltamivir (0.1 mg/ml), or suxiaoganmaojiaonang(4.375 mg/ml). The cultures were incubated at 37°C under 5% CO_2 _atm.

After 6, 12, 24, 36 and 48 h incubation in all antiviral assays, the supernatant was analyzed for the production of progeny virus using the hemagglutinin test and was compared with the untreated control cells. Cell proliferation and metabolism were analyzed by the CCK8-assay at 48 h post-treatment. Virus yields from the mock-treated cells were normalized to 100%.

### Real-time reverse transcription-PCR analysis

MDCK cells were grown to about 90% confluence infected with influenza virus (1 × 10^6 ^PFU). Medium was removed after 1 h, and cultured in the presence of dandelion extracts (2.5 mg/ml) 13 h. The inoculum was aspirated after 13 h. Cells were scraped off, washed twice with PBS, and collected by centrifugation (500 g for 5 min). Total RNA was prepared using the RNApure total RNA fast isolation kit (Shanghai Generay Biotech Co., Ltd). The primer sequence used for quantitative real-time PCR of viral RNA were 5' -TGTGTATGGACCTGCCGTAGC - 3' (sense) and 5' - CCATCCACACCAGTTGACTCTTG - 3' (antisense). The Canis familiaris beta-actin was used as internal control of cellular RNAs, with primer sequences of 5' -CGTGCGTGACATCAAGGAAGAAG - 3' (sense) and reverse: 5' -GGAACCGCTCGTTGCCAATG - 3' (antisense). The primer sequences used in real-time PCR were designed using Beacon Designer 7 software.

Real-time reverse transcription-PCR was performed using 100 ng of RNA and the One-step qPCR kit (RNA-direct SYBR Green Real-time PCR Master Mix, TOYOBO). Cycling conditions for real-time PCR were as follows: 90°C for 30 s, 61°C for 20 min, and 95°C for 1 min, followed by 45 cycles of 95°C for 15 s, 55°C for 15 s and 74°C for 45 s. As the loading control, we measured the level of Canis familiaris beta-actin mRNA. Real-time PCR was conducted using the ABI Prism 7300 sequence detection system, and the data were analyzed using ABI Prism 7300 SDS software (Applied Biosystems).

### Minigenome assay and transient transfection

To test the transcription efficiency of the influenza virus polymerases after drug treatment, a minigenome assay was performed in Human embryonic kidney (293T) cells. Briefly, ambisense plasmids encoding PB2, PB1, PA and NP were cotransfected together with the influenza virus replicon reporter plasmid pPOLI-luciferase. The reporter plasmid pPOLI-luciferase was constructed by inserting the luciferase protein open reading frame (ORF) flanked by the noncoding regions of the M gene of influenza A virus between the BamHIand NotI site of the pPOLI vector (a generous present from Dr. Edward Wright). Calcuim phosphate transfection was used. Briefly, the cell culture was replaced by Opti-medium; 0.5 μg of each plasmid was mixed, incubated at room temperature for 15 min, and added over 80% confluent 293T cells seeded the day before in six-well plates. Six hours later, the DNA-transfection mixture was replaced by DMEM containing 10% FBS. At 48 h posttransfection, the cells were treated with cell lysis buffer, centrifuged, and supernatant was collected. Add 5 μl aliquots of cell lysate to individual luminometer tubes containing 180 μl of luciferase assay buffer at room temperature. To start the assay, inject 100 μl of luciferin solution into the luminometer tube and measure the light output in the luminometer.

### Statistical analysis

Data were presented as mean ± SD. The data were statistically evaluated using a one-way ANOVA to compare differences between the groups. A p-value of < 0.05 was considered to be significant. The IC50 and CC50 values were calculated using GraphPad Prism programme.

## Results

### Treatment with aqueous dandelion extracts results in a reduction of progeny virus titers

**Figure 1:**
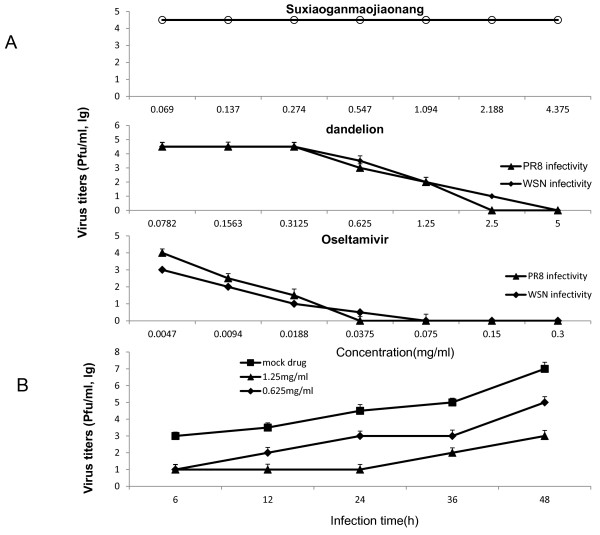
**Dandelion extracts inhibit influenza virus propagation**. Influenza virus (A/PR/8/34 [H1N1]) (1 × 10^6 ^PFU) were inoculated in MDCK cells. After 1 h, viruses were removed. (A) MDCK cells were treated with suxiaoganmaojiaonang (0.069-4.375 mg/ml), dandelion (0.0782-5 mg/ml), ostalmivir (0.0047-0.3 mg/ml) individually. The cultures were incubated for 24 h at 37°C under 5% CO_2 _atm. (B) MDCK cells were treated with dandelion (1.25 mg/ml, 0.625 mg/ml) individually. The cultures were incubated for 6, 12, 24, 36 and 48 h at 37°C under 5% CO_2 _atm. The yield of progeny viruses in MDCK supernatants was determined by plaque titrations assay. Each concentration of drugs was assayed two times in triplicate.

Treatment with aqueous dandelion extracts results in an efficient and concentration-dependent reduction of progeny virus titers in infected lung epithelial cells (A549) or Madine-Darby canine kidney (MDCK) cells; both of which are standard host cell lines for influenza virus propagation. These cells were treated with dandelion extracts at various concentrations (0.0782-5 mg/ml) 1 h post-infection with different influenza A virus strains, including human prototype isolate A/Puerto-Rico/8/34 (PR8) and A/WSN33 (WSN) (H1N1). The concentrations of the plant extract dilutions were kept constant in each sample throughout the experiment and showed a dose-dependent change in virus titer. Oseltamivir (0.0047-0.3 mg/ml) was used as a positive control and suxiaoganmaojiaonang (0.069-4.375 mg/ml) was used as a negative control for the inhibition of virus replication (Figure 1). The maximum inhibitory effect (100%) was obtained with 5 mg/ml, and the IC_50 _of dandelion extracts was 0.99 mg/ml.

### Dandelion treatment does not affect cell morphology, viability, or negatively interfere with proliferation and metabolism

**Figure 2:**
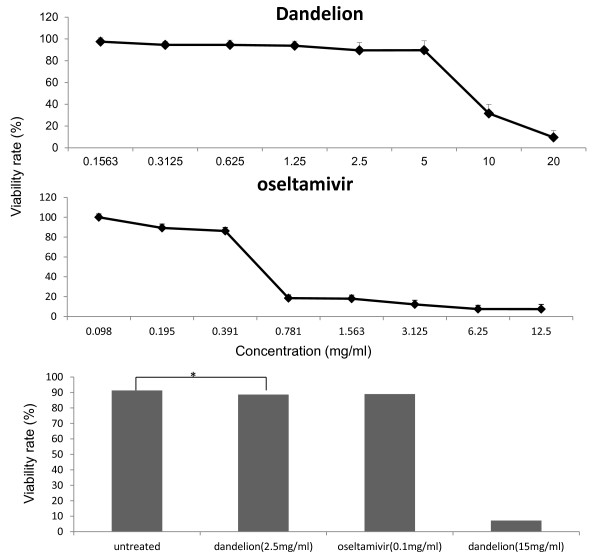
**Cytotoxicity assay of dandelion extracts**. (A) MDCK cells were left untreated (negative control) or treated with the indicated amounts of dandelion extracts or oseltamivir (2-fold dilutions) for 48 h. (B) MDCK cells were left untreated (negative control) or treated with 0.1 mg/ml oseltamivir, 2.5 mg/ml dandelion extracts and 15 mg/ml dandelion extracts (positive control) for 72 h. The cells were treated with CCK-8 solution (10 μl/well) and incubated for 4 h at 37°C. The absorbance was measured using a microplate reader at 450 nm. The untreated control was set at 100%. (* *p *> 0.05)

A major prerequisite for an antiviral agent is safety. Thus, we tested whether therapeutic concentrations of dandelion extracts would have any harmful effects on healthy cells. Initially, cells treated with dandelion extracts at the indicated concentrations were examined for changes in morphology. No differences in cell shape or cell number could be observed compared with untreated control cells. The same cells were treated with the CCK-8 solution to detect cell proliferation and metabolism in each sample (Figure 2). The CC_50 _of dandelion extracts was 8.47 mg/ml. SI = CC50/IC50 = 8.47/0.99 = 8.4.

### Inhibitory activity of dandelion extracts on influenza virus replication

**Figure 3:**
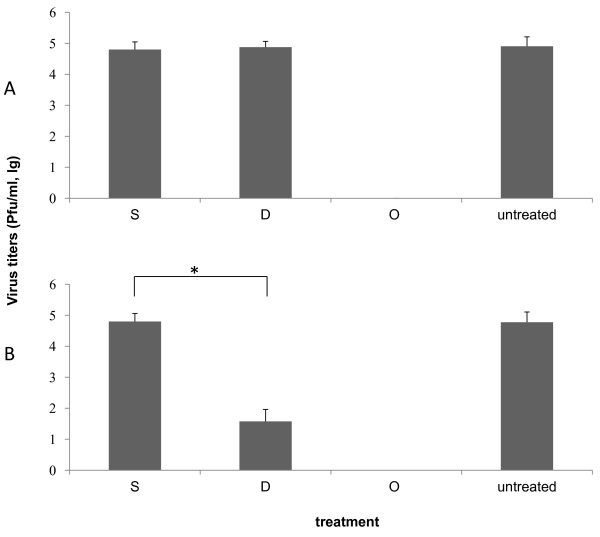
**Antiviral assay strategies with drugs on different stages of virus infection**. (A) Simultaneous treatment assay: MDCK cells were inoculated with PR8 treated with suxiaoganmaojiaonang (S, 4.375 mg/ml), dandelion (D, 2.5 mg/ml), ostalmivir (O, 0.3 mg/ml), or untreated with drugs (negative control) for 1 h, the media was removed and replaced by DMEM without any drugs; (B) Post treatment assay: Influenza viruses (1 × 10^6 ^PFU) were inoculated in MDCK cells. After 1 h, viruses were removed and MDCK cells were treated with suxiaoganmaojiaonang (S, 4.375 mg/ml), dandelion (D, 2.5 mg/ml), ostalmivir (O, 0.1 mg/ml) or untreated with drugs (negative control). The cultures were incubated for 16 h at 37°C under 5% CO_2 _atm. The yield of progeny viruses in MDCK supernatant was determined by plaque assay. Each concentration of drugs was assayed two times in triplicate.

The post treatment assay was performed to evaluate whether dandelion extracts are able to inhibit replication of influenza virus A/PR/8/34 and WSN (H1N1) in MDCK cells. Dandelion showed a strong antiviral activity against A/PR/8/34 and WSN (H1N1) at concentration 2.5 mg/mL (Figure 3B).

### Dandelion extracts does not block the hemagglutination activity of pre-treated virus particles

**Figure 4:**
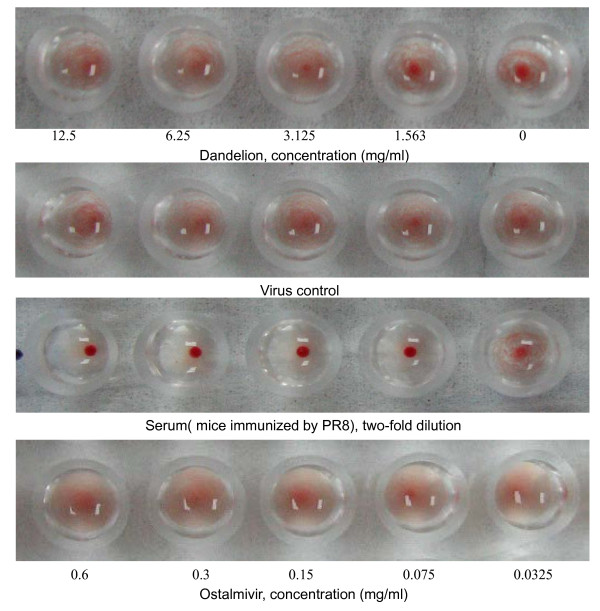
**Effect of dandelion extracts on agglutination with viral hemagglutinin and chicken RBC (cRBC)**. Four HAU of influenza virus (A/PR/8/34 [H1N1]) were mixed with an equal volume of 2-fold diluted dandelion extracts, ostalmivir (negative control), serum (mice immunized by PR8, positive control) or PBS (virus control) and incubated for 1 h at room temperature. The hemagglutination activity was tested by incubation with 1% (v/v) cRBC in PBS for 1 h at room temperature. We found dandelion extracts couldn't inhibit the viral hemagglutination.

To determine whether dandelion extracts would prevent the ability of virus particles to bind to cell surface receptors, we used simultaneous treatment assay and hemagglutination inhibition (HI) assays. The simultaneous treatment assay results indicated that treatment with dandelion extracts on virus entry couldn't inhibit virus infectivity (Figure 3A). Influenza A viruses are able to agglutinate red blood cells (RBCs) by means of hemagglutinin, a viral glycoprotein that binds to N-acetylneuraminic acid at the cell surface. The RBCs become cross-linked by the virus and will form a type of lattice. This cross-linking results in a diffuse distribution of the RBCs in a round-bottom vial, as compared with the spot-like appearance of RBCs in the absence of any virus. Pretreatment with dandelion extracts could not prevent the binding of different viruses to RBCs in this assay (Figure 4). These findings suggest that aqueous dandelion extracts do not block binding of viruses to cell receptors by directly interfering with viral HA.

### Viral RNA synthesis is affected in the treatment of dandelion extracts

**Figure 5:**
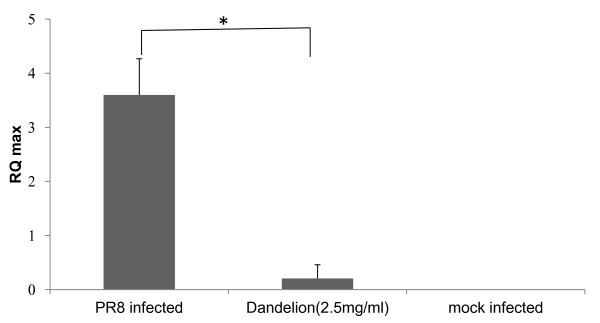
**Real-time reverse transcription-PCR of influenza viral Nucleoprotein (NP) RNA levels normalized to beta-actin**. MDCK cells were infected with influenza viruses A/PR/8/34 (H1N1) (1 × 10^6 ^PFU). After 1 h, viruses were removed. MDCK cells were treated with dandelion extracts (2.5 mg/ml) or untreated with drugs. Total RNA extraction was performed at 16 h after influenza virus infection and the levels of intracellular influenza viral RNA were measured. Influenza viral RNA levels normalized to beta-actin. (* *p *< 0.01). Mock-infected cells were also analyzed.

The levels of influenza viral RNA were compared between dandelion extracts -treated and untreated infected cells. RNA extraction was performed at 16 h after influenza virus infection and the levels of intracellular influenza RNA were measured. Quantitative real-time PCR showed a reduction of influenza RNA from dandelion extracts (2.5 mg/mL) treated cells comparison with the non-treated cells in both A/PR/8/34 (H1N1) and WSN. There were marked differences in NP RNA level between dandelion extracts-treated virus-infected cells and untreated virus-infected cells (Figure 5). These results indicate that blockage of virus replication is one of mechanisms, by which dandelion exerts antiviral effects.

### Treatment with dandelion extracts inhibit viral polymerase activity

**Figure 6:**
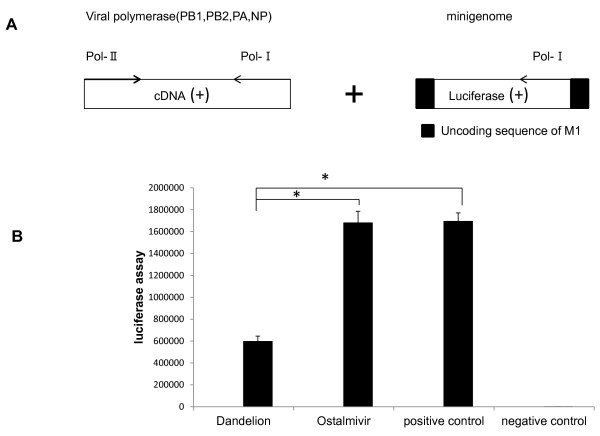
**Influence of drugs to the polymerase complex of A/PR8 virus strain**. (A) Scheme of the minigenome luciferase reporter assay. (B) The minigenome assay : HEK293T cells were transfected with the minigenome luciferase reporter assay without drug (positive control) or with dandelion extracts (2.5 mg/ml, ostalmivir (0.1 mg/ml). Mock-transfected cells were also analyzed as negative control. Cells were lysed after 48 h. The result was assayed by the luminometer. (* *p *< 0.01)

To evaluate if dandelion extracts influenced the polymerase activity, we performed a flu minigenome reporter assay (Figure 6A). The flu minigenome plasmid containing the luciferase reporter gene was cotransfected into 293T cells together with the four plasmids necessary for viral polymerase activity (PB2, PB1, PA and NP). The luciferase expression was quantified as described in Materials and Methods. There were marked differences between dandelion extracts treated virus-infected cells and non-treated or ostalmivir treated virus-infected cells (Figure 6B). These results indicate that dandelion inhibited the viral polymerase activity, then to exert antiviral effects.

## Discussion

Outbreaks of avian H5N1 pose a public health risk of potentially pandemic proportions. Infections with influenza A viruses are still a major health burden, and the options for the control and treatment of the disease are limited. Natural products and their derivatives have, historically, been invaluable sources of therapeutic agents. Recent technological advances, coupled with unrealized expectations from current lead-generation strategies, have led to renewed interest in natural products in drug discovery. This is also true in the field of anti-influenza research [24]. Here, we show that aqueous dandelion extracts exert potent antiviral activity in cell culture.

Dandelion is a natural diuretic that increases urine production by promoting the excretion of salts and water from the kidney. Dandelion extracts may be used for a wide range of conditions requiring mild diuretic treatment, such as poor digestion, liver disorders, and high blood pressure. Dandelion is also a source of potassium, a nutrient often lost through the use of other natural and synthetic diuretics. Additionally, fresh or dried dandelion herb is used as a mild appetite stimulant and to improve stomach symptoms, including feelings of fullness, flatulence, and constipation. The root of the dandelion plant is believed to have mild laxative effects and is often used to improve digestion.

Dandelion has a very high polyphenol content [18]. It is well known that polyphenols have protein-binding capabilities, which suggests that components of dandelion extracts may interact with pathogens through physical, non-specific interactions. Two potential advantages of this non-specific mechanism of action may be that resistant variants only emerge rarely and that dandelion extracts may also act against bacterial co-infections that represent a major complication in severe influenza virus infections. A non-specific interaction with viral HA has been reported for the polyphenolic compound epigallocatechin-gallate [17]. Simultaneous treatment was used to identify whether dandelion extracts block the viral adsorption to cells. The simultaneous treatment assay did not show significant antiviral activity (Figure 3A). These data indicate that dandelion extracts can not directly interfere with viral envelope protein at the cell surface. Therefore, we used HI assays to determine whether dandelion extracts interacted with HA of influenza virus (Figure 4). Dandelion extracts did not exhibit inhibition of viral HA in both A/PR/8/34 and WSN (H1N1), which agrees with the simultaneous treatment assay results.

To evaluate the anti-influenza activity after virus infection, we employed the post treatment assay (Figure 3B), quantitative real-time PCR (Figure 4) and minigenome assay (Figure 6) to test the in vitro effect of dandelion extracts on viral replication. Our studies do not show the prevention of receptor binding of the virus after dandelions treatment, but reduction of the nucleoprotein (NP) RNA level and the viral polymerase activity are obvious. Currently, anti-influenza targets include viral factors (such as hemagglutinin (HA), M2 ion channel protein, RNA-dependent RNA polymerase (RdRp), nucleoprotein (NP), non-structural protein (NS) and neuraminidase (NA) and host factors (such as v-ATPase, protease, inosine monophosphate dehydrogenase (IMPDH) and intracellular signalling cascades), and their relevant inhibitors [25]. In virus particles, the genomic RNAs (vRNAs) are associated with the RNA-dependent RNA polymerase proteins and the NP, which together form the ribonucleoprotein (RNP) complexes. The NP viral RNA level reflected the RNP complexes's action. Our results indicate that dandelion extracts inhibit influenza virus infection probably by decreasing the NP viral RNA level and viral polymerase activity, and thus affecting the RNP complexes' activities, further to inhibit viral RNA replication.

Vaccines play an important role in combating influenza. However, vaccination has only been able to provide a limited control of the infection, because the virus has a tendency to mutate and thus, escape the immune system. Plants have a long evolutionary history of developing resistance against viruses and have increasingly drawn attention as potential sources of antiviral drugs [24,26]. Many plant extracts and compounds of plant origin have been shown to possess activity against influenza viruses. Our results indicate that aqueous dandelion extracts can inhibit influenza virus infections. Dandelion is composed of multiple compounds that are able to regulate multiple targets for a range of medical indications and that are able to be titrated to the specific symptoms of an individual.

## Conclusion

This study has shown that dandelion extracts can inhibit both A/PR/8/34 and WSN (H1N1) influenza viruses by inhibiting viral nucleoprotein synthesis and polymerase activity. These results lead to further investigation about characterization of active compounds and their specific mechanism against influenza virus. Given the urgent need for new and abundantly available anti-influenza drugs, dandelion extracts appear to be a promising option as a replacement or supplemental strategy to currently available anti-influenza therapies.

## Competing interests

The authors declare that they have no competing interests.

## Authors' contributions

Conceived and designed the experiments: WH, BG. Performed the experiments: WH, HMH, WW. Contributed reagents/material/analysis tools: BG, WH, HMH, WW. Wrote the paper: WH, BG, HMH. All authors have read and approved the final manuscript.
